# GRP78-targeting subtilase cytotoxin sensitizes cancer cells to photodynamic therapy

**DOI:** 10.1038/cddis.2013.265

**Published:** 2013-07-25

**Authors:** M Firczuk, M Gabrysiak, J Barankiewicz, A Domagala, D Nowis, M Kujawa, E Jankowska-Steifer, M Wachowska, E Glodkowska-Mrowka, B Korsak, M Winiarska, J Golab

**Affiliations:** 1 Department of Immunology, Center of Biostructure Research, Medical University of Warsaw, 1A Banacha Street, F building, 02-097, Warsaw, Poland; 2 Department of Histology and Embryology, Center of Biostructure Research, Medical University of Warsaw, 02-004 Warsaw, Poland; 3 Department of Laboratory Diagnostics and Clinical Immunology of Developmental Age, Medical University of Warsaw, Marszalkowska 24, 00-576 Warsaw, Poland; 4 Department 3, Institute of Physical Chemistry, Polish Academy of Sciences, Warsaw, Poland

**Keywords:** GRP78, subtilase cytotoxin, cancer, photodynamic therapy

## Abstract

Glucose-regulated protein 78 (GRP78) is an endoplasmic reticulum (ER)-resident chaperone and a major regulator of the unfolded protein response (UPR). Accumulating evidence indicate that GRP78 is overexpressed in many cancer cell lines, and contributes to the invasion and metastasis in many human tumors. Besides, GRP78 upregulation is detected in response to different ER stress-inducing anticancer therapies, including photodynamic therapy (PDT). This study demonstrates that GRP78 mRNA and protein levels are elevated in response to PDT in various cancer cell lines. Stable overexpression of GRP78 confers resistance to PDT substantiating its cytoprotective role. Moreover, GRP78-targeting subtilase cytotoxin catalytic subunit fused with epidermal growth factor (EGF-SubA) sensitizes various cancer cells to Photofrin-mediated PDT. The combination treatment is cytotoxic to apoptosis-competent SW-900 lung cancer cells, as well as to Bax-deficient and apoptosis-resistant DU-145 prostate cancer cells. In these cells, PDT and EGF-SubA cytotoxin induce protein kinase R-like ER kinase and inositol-requiring enzyme 1 branches of UPR and also increase the level of C/EBP (CCAAT/enhancer-binding protein) homologous protein, an ER stress-associated apoptosis-promoting transcription factor. Although some apoptotic events such as disruption of mitochondrial membrane and caspase activation are detected after PDT, there is no phosphatidylserine plasma membrane externalization or DNA fragmentation, suggesting that in DU-145 cells the late apoptotic events are missing. Moreover, in SW-900 cells, EGF-SubA cytotoxin potentiates PDT-mediated cell death but attenuates PDT-induced apoptosis. In addition, the cell death cannot be reversed by caspase inhibitor z-VAD, confirming that apoptosis is not a major cell death mode triggered by the combination therapy. Moreover, no typical features of necrotic or autophagic cell death are recognized. Instead, an extensive cellular vacuolation of ER origin is observed. Altogether, these findings indicate that PDT and GRP78-targeting cytotoxin treatment can efficiently kill cancer cells independent on their apoptotic competence and triggers an atypical, non-apoptotic cell death.

Tumor microenvironment is characterized by insufficient vascularization, hypoxia, acidosis, increased glucose metabolism, and aberrant protein folding. All these conditions lead to upregulation of various components of the unfolded protein response (UPR), a signaling pathway triggered by endoplasmic reticulum (ER) stress.^1, 2^ Glucose-regulated protein 78 (GRP78) is an ER chaperone and a key regulator of the ER stress response signaling. Apart from its complex role in protein folding, it is involved in various stages of tumor progression. Upregulation of GRP78 correlates with higher pathological grade, recurrent disease, metastatic potential, and poor survival in many types of cancers.^3, 4^ GRP78 also has cytoprotective and anti-apoptotic roles contributing to chemo- and radioresistance.^5^

GRP78 is also induced in response to photodynamic therapy (PDT), a tumor treatment modality, which triggers oxidative stress and launches UPR.^6, 7^ After PDT, the misfolded proteins accumulate in the ER and increase the demand for proteins supporting folding machinery, including GRP78. Although the induction of GRP78 at mRNA as well as protein level had been demonstrated in various tumor models upon PDT, the general role of GRP78 upregulation in the cellular response to PDT is uncertain, as both increased sensitivity and enhanced resistance were reported depending on the photosensitizer (PS) or PDT protocol used. It was demonstrated that radiation-induced fibrosarcoma cells, when preincubated with Ca^2+^ ionophore A23187 to upregulate GRP78, are less sensitive to PDT in comparison with controls.^8^ In other studies, combination of the same^9^ or a different^10^ Ca^2+^ ionophore with PDT produced opposite results. A wide spectrum of effects triggered by Ca^2+^ ionophore treatment makes the interpretation of these discrepant results difficult. Moreover, it has been recently demonstrated that epigallocatechine gallate (EGCG), a GRP78-inhibiting agent, can improve the effectiveness of Photofrin-PDT (AxcanPharma Inc., Houdan, France).^11^ However, because of numerous pro-apoptotic activities induced by EGCG, it is impossible to delineate the role of GRP78 inhibition in the enhancement of PDT cytotoxicity.

Among the agents reported to downregulate or inhibit GRP78, which are characterized by a wide spectrum of additional activities, a notable exception is a bacterial cytotoxin SubAB, with a unique ability to specifically cleave GRP78.^12^ As the SubAB holotoxin is highly toxic and lethal to mice, a targeted delivery of subtilase cytotoxin catalytic A subunit (SubA) to tumor cells has been achieved by its fusion to a more specific targeting moiety, human epidermal growth factor (EGF).^13^ Epidermal growth factor receptor (EGFR)-targeted toxin (epidermal growth factor fused with subtilase cytotoxin subunit A; EGF-SubA) has been shown to selectively kill tumor cells at picomolar concentrations and to inhibit growth of human prostate cancer xenografts in mice. Furthermore, the EGF-SubA potentiated the cytotoxicity of ER stress-inducing agents.^13^ Apart from different mode of cell entry, the EGF-SubA caused GRP78 cleavage and ER stress induction, similar to the bacterial SubAB holotoxin.^13^

Using EGF-SubA for highly specific targeting of GRP78, we undertook an extensive study exploring the mechanisms of PDT-mediated induction of GRP78 and elucidating the role of this chaperone in PDT-induced cytotoxicity.

## Results

### GRP78 is upregulated in response to PDT and has a cytoprotective role

**Figure 1:**
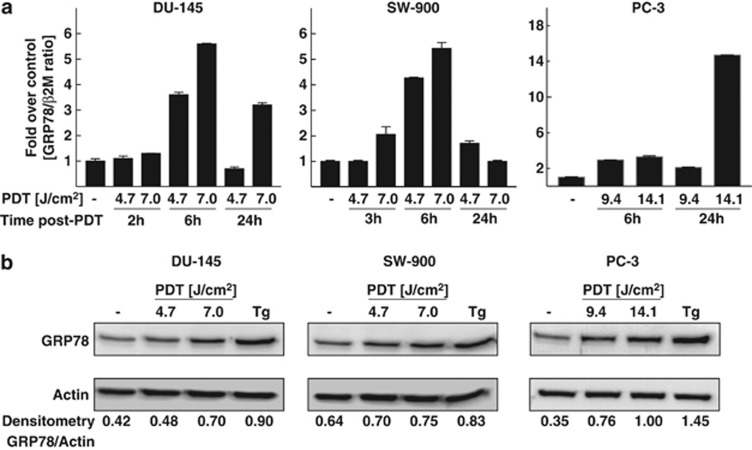
PDT induces GRP78 at mRNA (**a**) as well as at protein levels (**b**). (**a**) DU-145, SW-900 and PC-3 cells were subjected to *in vitro* PDT. At indicated time points after PDT, total RNA was isolated and reverse transcribed into cDNA. qPCR with LightCycler Fast Start DNA Master PLUS SYBRGreen I was performed to determine GRP78 mRNA and *β*_2_-microglobulin (B2M) levels. The figure presents mean fold over control change in experimental groups±S.D. (−) refers to controls. (**b**) Cells were subjected to *in vitro* PDT, collected 24 h after PDT and analyzed by western blotting for GRP78 and actin (loading control) expression. (−) refers to controls

Elevated GRP78 mRNA and protein levels were previously observed upon PDT with various PSs, however, mostly in murine cancer cell lines and *in vivo* in a murine experimental model.^8^ To investigate whether PDT induces GRP78 expression in human cancer cells, GRP78 mRNA and protein levels were evaluated in two prostate cancer cell lines: DU-145 and PC-3, and in a lung cancer cell line SW-900. As demonstrated in Figure 1a, dose-dependent increase in GRP78 mRNA was observed in all cell lines but with different kinetics. It is noteworthy that for each cell line, the kinetics of GRP78 mRNA induction was similar in response to thapsigargin (Supplementary Figure 1). The GRP78 protein levels were also elevated upon PDT, as measured with immunoblotting (Figure 1b).

**Figure 2:**
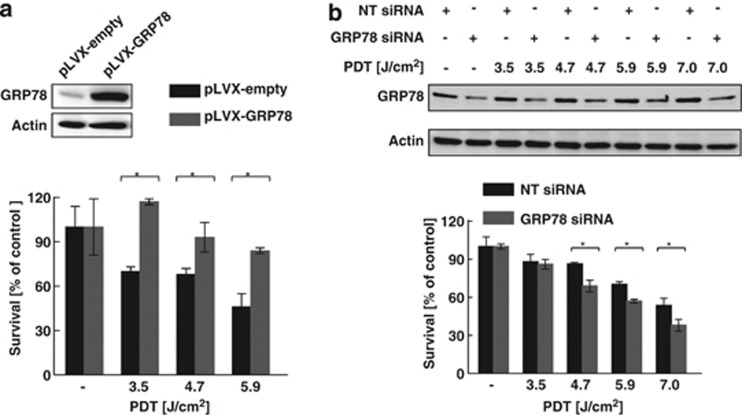
GRP78 level influences sensitivity of DU-145 cells to PDT. (**a**) DU-145 cells were infected with lentiviruses-containing human GRP78- and puromycin-resistant genes (*pLVX-GRP78-IRES-puro*) or puromycin-resistant gene only (*pLVX-IRES-puro*; mock control). Stable cell lines were isolated by means of incubation in the presence of 1 *μ*g/ml puromycin in culture medium. GRP78 overexpression was confirmed by western blotting (upper panel). The stable cell lines were subjected to *in vitro* PDT and cell survival was evaluated 24 h after PDT. For a particular stable cell line, the viability in each experimental group is calculated *versus* its own untreated control. The mean values from three independent measurements are shown, bars represent S.D., and *P<0.05 (Student's *t*-test). (−) refers to controls. (**b**) GRP78-specific or control siRNA at the final concentration of 20 nM was introduced to DU-145 cells via nucleofection. The cells were seeded into 35 mm dishes and incubated overnight with or without 10 *μ*g/ml Photofrin. After 24 h, cells were subjected to *in vitro* PDT. Twenty-four hours after PDT, the amount of GRP78 protein was evaluated by western blotting (upper panel) and cell viability was determined by crystal violet staining. The mean values from three independent measurements are shown, bars represent S.D., and *P<0.05 (Student's *t*-test). (−) refers to controls

To investigate the influence of GRP78 on PDT sensitivity, stable overexpression and RNAi approaches were used. A several-fold increase in GRP78 levels was observed in DU-145 cells infected with viruses containing GRP78 cDNA as compared with controls transduced with carrier vector. Cells stably overexpressing GRP78 were less susceptible to PDT at all tested PDT doses (Figure 2a). DU-145 cells with reduced, but still significant GRP78 levels, were slightly more sensitive to Photofrin-PDT at all tested light fluencies as compared with cells treated with non-targeting siRNA (Figure 2b, Supplementary Figure 2).

### EGF-SubA cytotoxin augments PDT-mediated cytotoxicity in various cancer cell lines

The above experiments demonstrated that GRP78 expression is induced upon Photofrin-PDT and contributes to lower PDT effectiveness. Therefore, a treatment modality combining PDT with GRP78-suppressing agent was considered. To avoid affecting multiple pathways with small-molecule GRP78 inhibitors, we used EGF-SubA,^13^ a targeted fusion toxin containing catalytic subunit A of subtilase cytotoxin, whose only target is GRP78.^12^ To examine cytotoxic effects of PDT+EGF-SubA, five different EGFR-positive human cancer cell lines were used: two prostate cancer cell lines (DU-145 and PC-3), a lung cancer cell line (SW-900), and two colon cancer cell lines (LoVo, HCT-116). As the occurrence of EGFR on the tumor cells is critical for EGF-SubA toxin delivery, its presence was confirmed on the surface of all selected cell lines (Supplementary Figure 3). Furthermore, as there are reports that PDT can affect EGFR expression,^14, 15^ the influence of Photofrin-PDT on EGFR level in DU-145 cells was evaluated. The results demonstrated dose-dependent, mild, and transient EGFR decrease upon Photofrin-PDT (Supplementary Figure 4). We concluded that EGF-SubA can be used for selective targeting of GRP78 in combination with Photofrin-PDT.

**Figure 3:**
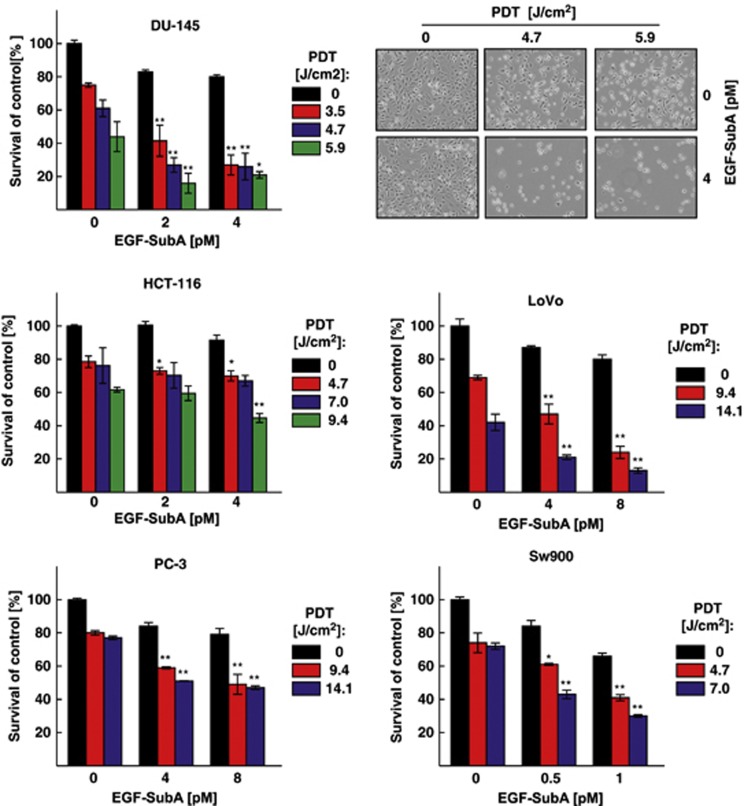
GRP78-targeting cytotoxin sensitizes various cancer cell lines to PDT. Human cancer cells were incubated for 24 h with 10 *μ*g/ml Photofrin and/or EGF-SubA cytotoxin for 24 h (DU-145, LoVo, and SW-900) or 48 h (PC-3, HCT-116) before exposure to different doses of light, as indicated on the right side of each graph. After additional 24 h of incubation, the cytotoxic effects were measured with crystal violet staining. The bars present percent survival relative to untreated controls. Data show mean values from two or three independent repeats±S.D., *P**<**0.05, **P**<**0.001 *versus* single modality-treated cells (EGF-SubA or PDT only). Upper right panel demonstrates representative phase-contrast microscopic photographs of DU-145 control cells and cells treated with EGF-SubA, PDT or the combination of EGF-SubA and PDT at indicated PDT and EGF-SubA doses

Treatment protocols were optimized for each cell line. In all cell lines, the treatment regimen including both PDT+EGF-SubA turned out to be the most efficient (Figure 3). Chou and Talalay^16^ calculations using a CalcuSyn software (Biosoft) revealed that the combination of PDT and EGF-SubA exerts synergistic cytotoxic effects against DU-145, PC-3, LoVo, and SW-900, but not HCT-116 cells.

**Figure 4:**
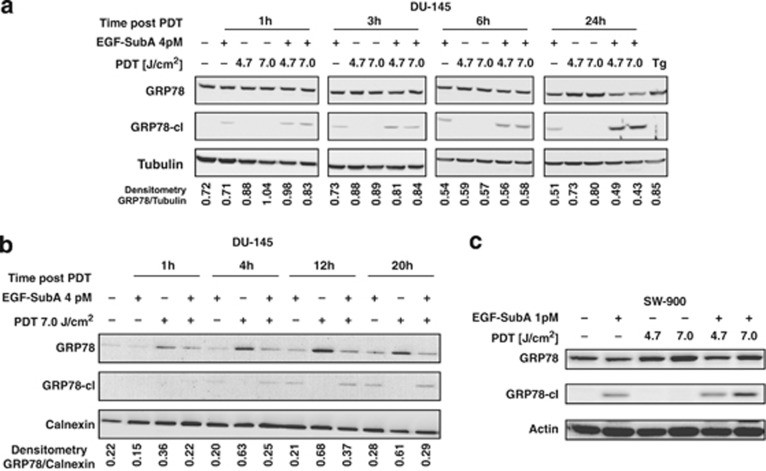
EGF-SubA cytotoxin cleaves PDT-upregulated GRP78 and generates 46 kDa cleavage product. (**a**) DU-145 cells were seeded into 60 mm culture dishes and the next day EGF-SubA cytotoxin was added to indicated samples. After additional 24 h, cells were subjected to *in vitro* PDT and further cultured in full medium alone or with EGF-SubA for indicated time (1, 3, 6, or 24 h). Total cell lysates were analyzed by western blotting with anti-GRP78 antibody (Cell Signaling, recognizes GRP78 N-terminal domain) for the level of full-length GRP78 protein and 46 kDa GRP78 cleavage product generated by EGF-SubA cytotoxin (GRP78-cl) and with anti-tubulin antibody (loading control). The experiment was repeated several times and the representative result is shown. The ratio of full-length GRP78 to tubulin was calculated using GelAnalyzer 2010 software and the number is given below each lane. Tg refers to sample treated with 10 *μ*M thapsigargin for 24 h (positive control for GRP78 overexpression).(**b**) The experiment was performed as in **a**, but at indicated times after PDT the membrane fraction was isolated with Subcellular Protein Fractionation Kit (Thermo Scientific) and analyzed by western blotting. The ratio of full-length GRP78 to calnexin was calculated using GelAnalyzer 2010 software and the number is given below each lane. (**c**) SW-900 cells were subjected to *in vitro* PDT in combination with EGF-SubA cytotoxin, according to the protocol described in Materials and Methods section. Twenty-four hours after PDT, total cell lysates were analyzed by western blotting to detect the levels of GRP78 and its 46 kDa cleavage product as described in **a**

Next, the levels of full-length GRP78 protein and its cleavage fragment generated by EGF-SubA cytotoxin were investigated in whole-cell lysates in a time course after PDT in DU-145 cell line. Intact GRP78 protein was slightly upregulated as early as 1 h after PDT and reached the maximum at 24 h after PDT. Addition of EGF-SubA resulted in downregulation of intact GRP78 and gradual accumulation of the cleavage fragment (Figure 4a). Remarkably, the intact GRP78 induction upon PDT and its downregulation by EGF-SubA cytotoxin were more prominent when the lysates of membrane/organelles fraction only were analyzed with immunoblotting (Figure 4b). Downregulation of intact GRP78 protein and generation of its cleavage fragment with EGF-SubA were also observed in SW-900 cells in response to the combination treatment (Figure 4c).

Altogether, these data demonstrate a significant potentiation of PDT-mediated cytotoxicity by EGF-SubA cytotoxin accompanied by GRP78 cleavage and downregulation.

### EGF-SubA cytotoxin enhances PDT-mediated UPR

**Figure 5:**
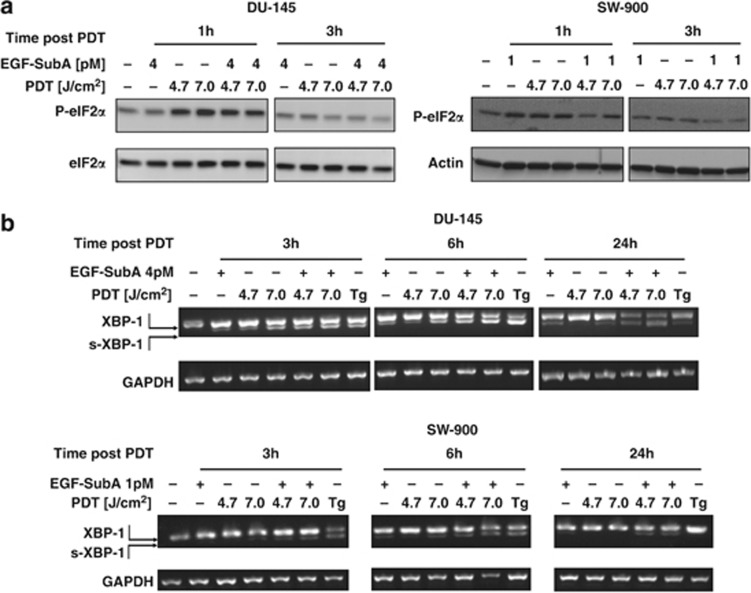
EGF-SubA dysregulates PDT-induced ER stress. (**a**) DU-145 (left panel) or SW-900 cells (right panel) were subjected to *in vitro* PDT in the presence or absence of indicated concentration of EGF-SubA, and whole-cell lysates were collected 1 and 3 h after PDT. The level of phosphorylated eIF2*α* (P-eIF2*α*) was estimated by western blotting. Unphosphorylated eIF2*α* (DU-145 cells) or actin (SW-900 cells) were used as loading controls. (**b**) Cells were treated as in **a**, but at indicated times after PDT the cells were lysed, total RNA was isolated, reverse transcribed to cDNA and PCR was performed to detect spliced (s-XBP-1) and unspliced (XBP-1) forms of XBP-1. Glyceraldehyde 3-phosphate dehydrogenase (GAPDH) was used as a loading control. PCR products were separated on 2% agarose gel and visualized by incubation in 0.5 *μ*g/ml ethidium bromide solution

GRP78-regulated UPR signaling pathways in response to the combined therapy were then investigated in DU-145 and SW-900 cell lines. The translation control arm of the UPR was assessed by monitoring the phosphorylation of eukaryotic translation initiation factor 2 subunit alpha (eIF2*α*) on serine 51, a commonly used indicator of protein kinase R-like ER kinase activation. Increased eIF2*α* phosphorylation was observed 1 h after PDT in both cell lines. EGF-SubA cytotoxin slightly attenuated eIF2*α* phosphorylation in DU-145 cells and significantly in SW-900 cells. The phosphorylation was transient and at 3 h after PDT it returned to the control level (Figure 5a). The activation of inositol-requiring enzyme 1 was examined by the detection of alternative X-box-binding protein 1 (XBP-1) transcript formation with reverse transcription (RT)-PCR. Results for both cell lines were similar. In cells undergoing PDT alone, the presence of the shorter, spliced variant of XBP-1 (s-XBP-1) was observed at 3 and 6 h after PDT, but not at 24 h after PDT. In cells treated with PDT+EGF-SubA, the level of the s-XBP-1 was further increased at 3 and 6 h after PDT, and it was maintained at 24 h after PDT (Figure 5b).

### ER stress induced by EGF-SubA and PDT leads to caspase activation, but is not the major trigger of the combined therapy-induced cell death

**Figure 6:**
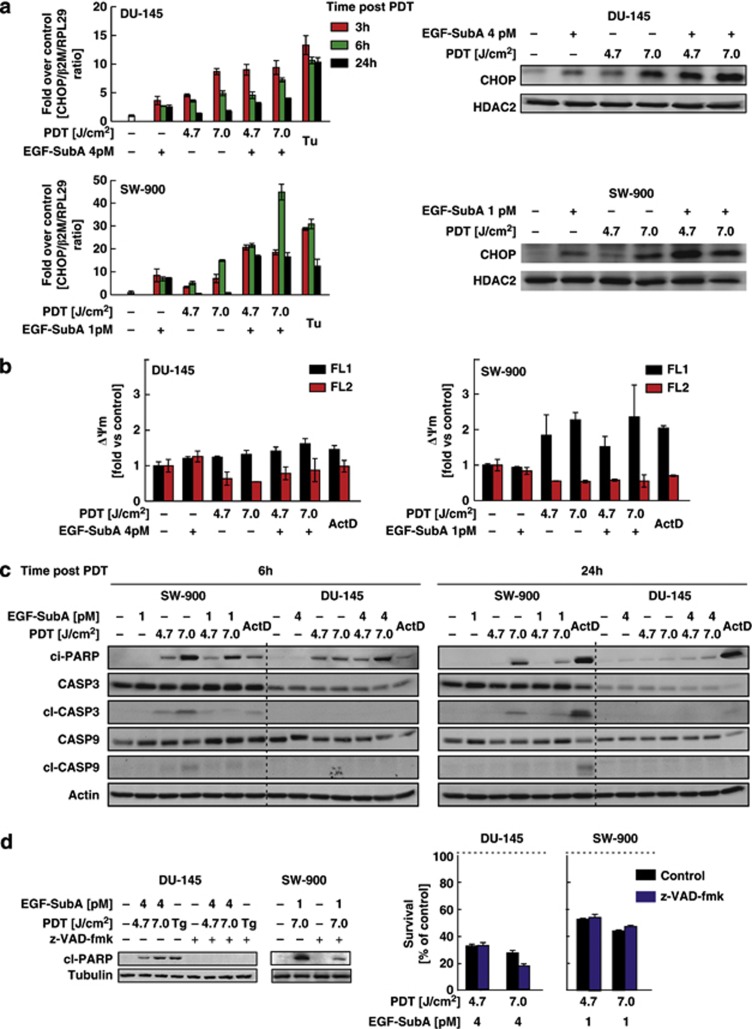
PDT and EGF-SubA cytotoxin induce early stages of apoptosis. DU-145 and SW-900 cells were subjected to *in vitro* PDT in the presence or absence of EGF-SubA, to 10 *μ*M Tg, or to 1 *μ*g/ml actinomycin D, and collected at various time points after PDT. (−) refers to controls. (**a**) left panel: at indicated times after PDT, total RNA was isolated and reverse transcribed into cDNA. qPCR with LightCycler Fast Start DNA Master PLUS SYBRGreen I was performed to determine CHOP, B2M, and ribosomal protein L29 (RPL29) mRNA levels. The figure presents mean fold over control change in experimental groups±S.D. Tu refers to samples treated with 10 *μ*g/ml of tunicamycin. (**a**) right panel: a nuclear soluble fraction of cells collected 20 h after PDT was isolated with Subcellular fractionation kit (Thermo Scientific), and the level of CHOP and HDAC2; loading control) was evaluated by western blotting. (**b**) Four hours after PDT, the changes in mitochondrial membrane potential were quantified with MitoLight (Millipore). Green fluorescence of the dye monomers applies to apoptotic cells and red fluorescence of the dye J-aggregates applies to healthy cells with polarized mitochondria. The figure shows the mean fluorescence values normalized to untreated control±S.D. ActD refers to samples treated with 1 *μ*g/ml of actinomycin D for 24 h. (**c**) Whole-cell lysates were collected 6 and 24 h after PDT, and the levels of cleaved PARP, caspase-3 and -9 and their activated, cleaved forms were analyzed by western blotting. ActD refers to samples treated with 1 *μ*g/ml of actinomycin D for 6 or 24 h. (**d**) left panel: cleaved PARP and tubulin levels were analyzed by western blotting as in **c**, 24 h after PDT. For indicated samples, 2 h before PDT and after PDT z-VAD-fmk was added to the culture medium at the final concentration of 100 *μ*M. (**d**) right panel: cell viability in the absence (control) and presence of 100 *μ*M z-VAD-fmk was determined by crystal violet staining. Dotted line indicates the survival of control cells (100%)

It is well documented that prolonged or exaggerated ER stress leads to cell death. Considering the induction of UPR signaling pathways in response to PDT and their dysregulation by EGF-SubA, the link between UPR and cell death was examined. C/EBP (CCAAT/enhancer-binding protein) homologous protein (CHOP) is the central transcription factor upregulated during UPR that is considered a major trigger of ER stress-induced apoptosis. PDT increased the levels of CHOP mRNA, as measured with quantitative real-time PCR (qPCR), and this effect was further enhanced by EGF-SubA (Figure 6a, left panel). Consistently, CHOP protein accumulated in the nucleoplasm of cells after PDT and to even more extent upon PDT+EGF-SubA cytotoxin, as shown in Figure 6a, right panel.

To test whether apoptosis was induced in response to PDT+EGF-SubA in Bax-deficient DU-145 cells and in wild-type Bax SW-900 cells, various early and late apoptosis markers were investigated. SW-900 cells treated with PDT and PDT+EGF-SubA lost their mitochondrial membrane integrity in the first 4 h after PDT. Despite Bax deficiency, some disruption of mitochondrial membrane potential could also be detected in PDT-treated DU-145 cells, although the effect was much weaker, even after treatment with a common apoptosis inducer actinomycin D (Figure 6b). This was followed by the release of cytochrome c into the cytoplasm (Supplementary Figure 5). Next, the activation of caspases-3 and -9 was investigated with immunoblotting. In SW-900 cells, cleaved caspases-3 and -9 were detected in PDT-treated cells 6 h after PDT, but their amounts were noticeably lower when EGF-SubA was combined with PDT. In DU-145 cells, cleaved caspases were not observed. Although cleaved poly (ADP-ribose) polymerase (PARP), a product of caspase cleavage, was detected in both cell lines upon PDT, the EGF-SubA cytotoxin decreased the amount of cleaved PARP in SW-900 cells but increased the amount of cleaved PARP in DU-145 cells (Figure 6c). In the presence of pan-caspase inhibitor z-VAD, the generation of cleaved PARP could be completely eliminated or significantly reduced in DU-145 and SW-900 cells, respectively (Figure 6d, left panel). However, z-VAD did not influence the cytotoxicity of the combination therapy in any of the cell lines (Figure 6d, right panel).

**Figure 7:**
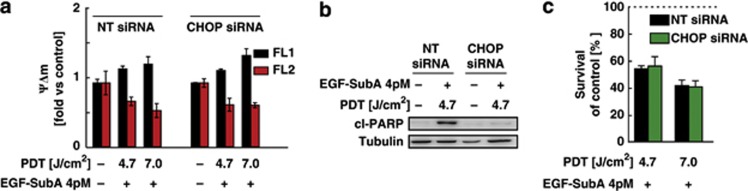
CHOP does not contribute to PDT and EGF-SubA cytotoxic effect. Forty-eight hours before PDT, CHOP-specific or control siRNA at the final concentration of 50 nM was introduced to DU-145 cells via nucleofection. (**a**) Four hours after PDT, the changes in mitochondrial membrane potential were quantified with MitoLight (Millipore), as described in Figure 6b. The figure shows the mean fluorescence values normalized to untreated control±S.D. (**b**) Twenty-four hours after PDT, cells were collected and 15 *μ*g of whole-cell lysates was analyzed by western blotting to assess the levels of cleaved PARP and tubulin (loading control). (**c**) Cell viability was determined by crystal violet staining, 24 h after PDT. Dotted line indicates the survival of control cells (100%)

Next, we investigated the role of CHOP in cell death mediated by PDT+EGF-SubA in DU-145 cells. CHOP silencing with siRNA was efficient (Supplementary Figure 6), but it did not prevent the disruption of mitochondrial membrane potential triggered by PDT+EGF-SubA (Figure 7a). Although CHOP silencing reduced caspase activation in response to the combination therapy as indicated by the decreased PARP cleavage (Figure 7b), it did not significantly influence the sensitivity of DU-145 cells to the therapy (Figure 7c). Moreover, despite induction of CHOP, mitochondrial depolarization, and PARP cleavage, later apoptotic events such as phosphatidylserine plasma membrane externalization and DNA fragmentation were not reinforced in response to the combination treatment in any of the investigated cell lines (Supplementary Figure 7).

Altogether, these results indicate that apoptosis is not the major cell death mode responsible for the combined therapeutic toxicity neither in apoptosis-deficient DU-145 nor in apoptosis-competent SW-900 cells.

### EGF-SubA cytotoxin augments cellular vacuolation induced by PDT

Despite lack of typical features of late-stage apoptosis, the combination of EGF-SubA and PDT was toxic both to DU-145 and SW-900 cells. Therefore, other modes of cell death were considered. Although the morphological features of the cell death did not resemble necrosis, we examined the plasma membrane integrity of DU-145 cells treated with PDT or PDT+EGF-SubA cytotoxin, in a time course after PDT. As measured by lactate dehydrogenase release, the plasma membrane integrity was maintained as long as 16 h after PDT. Furthermore, treatment with necrostatin-1, which suppresses some forms of necrotic cell death, failed to inhibit PDT+EGF-SubA-induced cell death (Supplementary Figure 8).

It was shown that porphycene-PDT can induce autophagic cell death in Bax-deficient DU-145 cells^17^ and that it can be suppressed by the phosphoinositide 3-kinase (PI3K) inhibitors. On the other hand, it was demonstrated that GRP78 is indispensable for ER stress-mediated autophagy induction.^18^ Considering these facts, we have investigated whether autophagy is induced in response to Photofrin-PDT or PDT+EGF-SubA. Remarkably, typical hallmarks of autophagy were not detected in DU-145 cells undergoing PDT alone or, PDT+EGF-SubA. Moreover, markers of autophagy were undetectable after starvation, a typical autophagy inducer, nor after incubation with chloroquine, a lysosomotropic agent preventing fusion of endosomes with lysosomes. Instead, accumulation of light chain 3, microtubule-associated protein (LC3-I) was observed in cells undergoing PDT, PDT+EGF-SubA, or treatment with chloroquine, indicating a defect of autophagy in these cells (Supplementary Figure 9A). These results are in agreement with the recent report showing that DU-145 cells are autophagy deficient because of mutations in *ATG5* gene.^19^ Intriguingly, the cytotoxicity of PDT and PDT+EGF-SubA could be significantly delayed, but not inhibited, by a PI3K inhibitor 3-methyladenine (3-MA; Supplementary Figure 9B), and only slightly delayed with another PI3K inhibitor wortmannin (WA; not shown).

**Figure 8:**
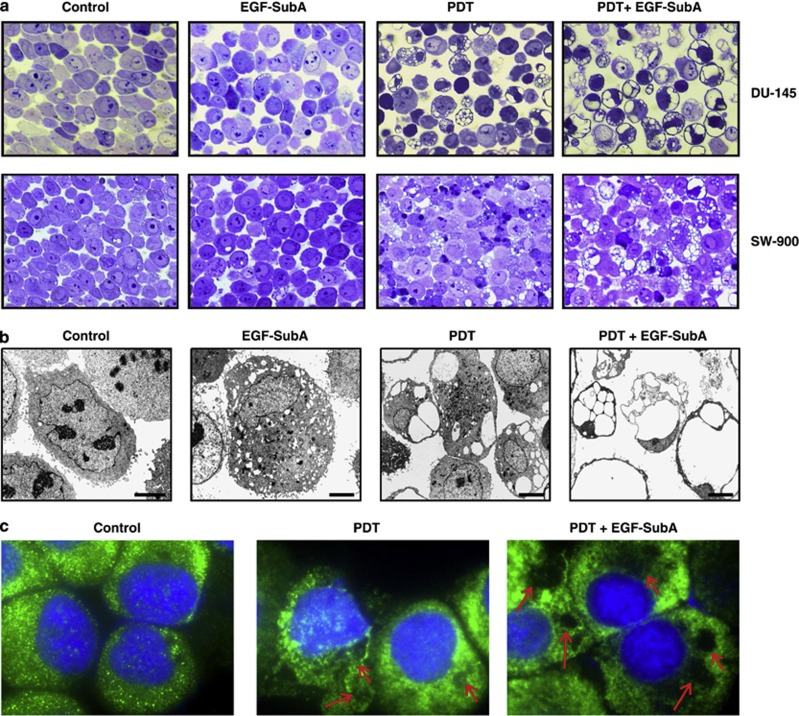
PDT and EGF-SubA cytotoxin trigger massive cytoplasmic vacuolation. (**a**) Control and treated cells were collected by trypsinization 10 h after PDT (DU-145 cells, upper panel) or 16 h after PDT (SW-900 cells, lower panel), pelleted and washed twice with PBS. Next, pellets were fixed and dehydrated using standard preparation (described in Materials and Methods section), cut on semithin sections, stained with toluidine blue, and inspected in Eclipse E400 light microscope using × 40 objective lens. (**b**) DU-145 cells were processed for electron microscopy as described in the Materials and Methods section, and 60–90 nm ultrathin sections, after staining with uranyl acetate and lead citrate, were examined under a transmission electron microscope. Scale bars represent 5 *μ*m. (**c**) Ten hours after PDT, control and treated DU-145 cells were collected and cytospun onto microscopic slides. Cells were stained for calnexin using anti-calnexin primary antibody (1 : 50, 1% BSA/PBS+0.3% Triton x-100) and subsequently with Alexa Fluor 488 Donkey Anti-Rabbit secondary antibody (1 : 100, 1% BSA/PBS+0.3% Triton x-100). Cell nuclei were stained with DAPI. Cells were analyzed under the fluorescent microscope.

Lack of any typical features of apoptosis, necrosis, or autophagy prompted a more detailed examination of cells undergoing PDT or PDT+EGF-SubA with light microscopy of fixed, semithin, toluidine blue-stained sections. An extensive cellular vacuolation was observed after PDT in DU-145 and SW-900 cells, and it was significantly intensified by EGF-SubA cytotoxin (Figure 8a). A more detailed, electron microscopic analysis was performed only for DU-145 cells. The vacuoles were of different size and number, ranging from numerous and small (Supplementary Figure 10B) to less numerous and larger (Supplementary Figure 10C), as well as huge, filling almost the entire cell (Supplementary Figure 10D). Large vacuoles were empty, however, some small, round-shaped vacuoles contained remnants of homogeneous material of moderate electron density, suggesting lipid content. Some vacuoles arose from strongly distended perinuclear cisternae (Supplementary Figure 10E; black arrows), which are considered to be parts of rough ER. Remarkably, the vacuolation was intensified in cells treated with PDT+EGF-SubA (Figures 8a and b). This was expressed as an increase in the number of affected cells and as a higher degree of vacuolation (more cells with large vacuoles present). In some cells vacuolation was not prominent. However, in these cells (Supplementary Figure 10A), as well as in the vacuolated cells (Supplementary Figures 10B and C), numerous dark, condensed, degenerated mitochondria, and lysosomes were present. Seldom, isolation membranes surrounding a portion of the cytoplasm or organelle were observed (Supplementary Figure 10F; white arrows). Such images may suggest an early phase of autophagy.

In order to investigate the subcellular origin of the vacuoles, immunostaining for calnexin, the ER membrane protein, was done. In control DU-145 cells, the staining for calnexin was even throughout the cytoplasm, which is characteristic of a typical ER membrane staining. However, in cells treated with PDT+EGF-SubA cytotoxin, calnexin immunostaining concentrated around unstained, round-shaped regions, a pattern consistent with the presence of vacuoles in these cells (Figure 8c). The atypical, non-apoptotic cell death with peculiar extensive cytoplasmic vacuolation has been previously observed in DU-145 cells upon proteasome inhibition with MG-132.^20^

## Discussion

The major finding of this report is that the efficacy of Photofrin-mediated PDT against cancer cells can be enhanced by a highly selective destruction of GRP78 with a targeted subtilase cytotoxin EGF-SubA. Previous studies indicated that PDT with various photosensitizers leads to GRP78 upregulation in tumor cells.^8, 10^ Although still contentious to some extent, the majority of observations indicate that increased GRP78 levels have a cytoprotective role and have the potential to regulate UPR signaling to mitigate PDT-induced ER stress.^1, 4^ In many cell types, overexpression of GRP78 reduces apoptosis, mitigates cytosolic Ca^2+^ overload induced by ER stressors, and confers increased chemo- and radioresistance.^5, 21^ Not surprisingly, an increased GRP78 expression seems to correlate with tumor recurrence and decreased survival, making this chaperone a promising biomarker of treatment outcome and a potential therapeutic target.^1, 22, 23, 24^ For example, EGCG-induced downregulation of GRP78 levels was shown to improve PDT efficacy in murine mammary carcinoma cells.^11^ However, mechanistic interpretation of these results is difficult because, in addition to GRP78, the targets of EGCG include a number of other PDT-inducible molecules including matrix metalloproteinases, cyclooxygenase 2, hypoxia-inducible factor, and many signal transduction pathways, which have been involved in the regulation of PDT-mediated cytotoxicity in tumor cells.^25^ Therefore, more selective approaches to targeting GRP78 are necessary to delineate the role of this chaperone in response to PDT. A bacterial cytotoxin SubAB has an unusual ability to specifically cleave GRP78 without affecting other proteins in the cell.^12^ Although its potential use in the treatment of cancer is unattainable because of systemic toxicity associated with promiscuous binding of B-subunit, a targeted delivery of the catalytic subunit A makes therapeutic approaches more feasible. Indeed, a fusion toxin EGF-SubA was shown to be cytotoxic to EGFR-expressing cells.^13^ Therefore, we employed EGF-SubA to test whether it can potentiate antitumor efficacy of Photofrin-PDT.

Detailed analysis of cellular responses to Photofrin-PDT revealed that this treatment increases GRP78 expression (Figures 1a and b). Although GRP78 overexpression was associated with increased resistance of tumor cells to PDT-mediated cytotoxicity (Figure 2A), its incomplete downregulation slightly, but significantly reduced viability of PDT-treated cells (Figure 2b). EGF-SubA significantly potentiated PDT-mediated cytotoxicity in five different human tumor cell lines, which was accompanied by a specific GRP78 cleavage (Figures 3 and 4). Intriguingly, the level of non-cleaved GRP78 remained high, indicating that *de novo* synthesis of GRP78 is continued (Figures 4a–c). In previous studies, partial inhibition of GRP78 expression, through antisense or RNA interference approaches, did not affect the survival of tumor cells.^26, 27^ However, SubA-induced cleavage of GRP78 led to cell death.^12^ A number of mechanisms can be considered to explain significant activity of EGF-SubA despite incomplete GRP78 downregulation. For example, rapid activation of ER stress-associated signaling pathways resulting from GRP78 cleavage can trigger robust response inefficiently compensated by slower upregulation of GRP78 expression or by other protective pathways. These mechanisms of potentiated cytotoxicity seemed likely, considering that PDT triggers oxidative stress resulting in protein unfolding and activation of ER stress. PDT induced a rapid but rather transient phosphorylation of eIF2*α*, which was slightly attenuated after incubation of tumor cells with EGF-SubA, suggesting that restoration of translation by the cytotoxin may contribute to potentiation of the cytotoxic effect of PDT (Figure 5a).

It should be emphasized that DU-145 cells are Bax-deficient and resistant to apoptosis triggered by various therapeutic approaches.^28^ Although the combination of PDT+EGF-SubA resulted in mitochondrial membrane depolarization (Figure 6b) followed by cytochrome c release (Supplementary Figure 5) and increased PARP cleavage (Figure 6c), DU-145 cells did not reveal typical hallmarks of apoptosis such as PS externalization or DNA fragmentation (Supplementary Figure 7). Despite effective inhibition of PARP cleavage, z-VAD, a pan-caspase inhibitor, did not suppress cytotoxicity of the combined treatment (Figure 6d). Similar observations were made in SW-900 cells, which have a functional Bax, and can be induced to undergo apoptosis. PDT induced apoptosis in these cells, but, despite potentiating PDT-mediated cytotoxicity, EGF-SubA cytotoxin diminished caspase-3 and -9 activation (Figure 6c) and PS externalization (Supplementary Figure 7). As CHOP downregulation with siRNA failed to ameliorate PDT+EGF-SubA toxicity, despite preventing PARP cleavage, it does not seem to be directly involved in the treatment outcome (Figure 7). However, only recently it has been shown that the joint action of activation transcription factor (ATF)4 and CHOP causes restoration of protein synthesis and promotes cell death, and ATF4 has a primary role.^29^ Further investigation is needed to elucidate the role of ATF4 in the PDT+EGF-SubA-triggered cell death.

Also, no features typical for necrotic cell death were observed in DU-145 cells undergoing PDT in combination with EGF-SubA cytotoxin. Next to the lack of morphological features of necrosis, the plasma membrane integrity was maintained in DU-145 cells (Supplementary Figure 8A) and necrostatin-1, which suppresses some forms of necrotic cell death, was ineffective in inhibiting PDT+EGF-SubA-induced cell death (Supplementary Figure 8B).

Embryonic fibroblasts from Bax/Bak double knockout mice are resistant to apoptosis but still undergo a non-apoptotic death after death stimulation, which is associated with autophagosomes/autolysosomes.^30^ Although GRP78 is required for stress-induced autophagy to occur,^18^ we investigated whether the combination of PDT+EGF-SubA cytotoxin can trigger autophagy and/or autophagy-associated cell death, as previous studies revealed that PDT can induce autophagy in DU-145 cells.^17^ Cytotoxic activity of PDT+EGF-SubA was only delayed but not inhibited by PI3K inhibitors 3-MA or WA that block an early stage of autophagy by inhibiting the class III PI3K (Supplementary Figure 9). There was no LC3 processing to its activated form, but the combined regimen led to an increase in LC3-I levels (Supplementary Figure 9). This observation is consistent with a recent report indicating that DU-145 cells are autophagy deficient because of mutations in *ATG5* gene.^19^ Also, TEM of DU-145 did not reveal ultrastructural alterations indicating formation of autophagic compartments. However, a robust cellular vacuolation was observed after PDT, which was exacerbated by the addition of EGF-SubA cytotoxin (Figure 8). Also, in apoptosis-competent SW-900 cells, increased cytoplasmic vacuolation was readily observed after combination treatment. Immunostaining revealed that the vacuoles are of ER origin, with intensive calnexin staining (Figure 8C). Similar morphological changes were observed in Bax-deficient DU-145 cells in response to proteasome inhibition of MG-132,^20^ or incubated with 15-deoxy-Δ^12, 31^-prostaglandin J_2_,^32^ as well as in cells with activated insulin-like growth factor I receptor.^33^

Altogether, the studies reported here indicate that the combination of PDT with EGF-SubA cytotoxin generates synergistically enhanced cytotoxicity toward tumor cells, and in two investigated cell lines it leads to an unusual type of tumor cell death accompanied by massive cytoplasmic vacuolation. As EGFR overexpression is associated with highly aggressive and metastatic tumor cells,^34, 35^ EGF-SubA+PDT combination might be particularly effective against primary invasive tumors either alone or in an adjuvant setting to surgery.

## Materials and Methods

### Reagents

All chemicals and other reagents were purchased from Sigma-Aldrich (St Louis, MA, USA), unless stated otherwise. Photofrin (porfimer sodium), a PS used for all experiments, was dissolved in Dulbecco's modified Eagle's medium to make a 0.5 mg/ml stock solution, aliquoted, and stored at −80^o^C. Construction and purification of EGF-SubA fusion protein (SibTech, Inc. Brookfield, CT, USA) has been described previously.^13^ The 7.5 *μ*M stock solution was aliquoted and stored at −80^o^C. z-VAD-fmk, Tg, and tunicamycin were from Santa Cruz Biotechnology (Santa Cruz, CA, USA).

### Cell culture

Human prostate cancer cell lines PC-3 and DU-145, human colorectal carcinoma cell lines HCT-116 and LoVo, and human squamous cell lung carcinoma SW-900 were purchased from the American Type Culture Collection (Rockville, MD, USA). Cells were cultured in Dulbecco's modified Eagle's medium (DU-145, SW-900, PC-3), RPMI 1640 (LoVo), or Dulbecco's modified Eagle's medium F12-HAM (HCT-116), supplemented with 5% (DU-145) or 10% (other cell lines) heat-inactivated fetal bovine serum and antibiotic/antimycotic solution (Sigma-Aldrich). Cells were maintained at 37 °C, 5% CO_2_, in a humidified atmosphere.

### *In vitro* PDT

Cells were seeded into 35- or 60-mm culture dishes at a density of ∼1 × 10^4^ cells/cm^2^ and were allowed to attach overnight. Photofrin (10 μg/ml) was added and cells were further incubated in the dark for 24 h. Before light exposure, the medium in each plate was replaced with PBS and cells were illuminated with white light generated by 100 W sodium lamp through a red filter. After illumination, cells were incubated for additional 24 h in fresh medium. For combination experiments, EGF-SubA cytotoxin was added to the culture medium 48 h (PC-3, HCT-116) or 24 h (all other cell lines) before light exposure and then after light exposure for remaining 24 h, at the indicated concentrations.

### Cell viability assays

To evaluate cytostatic/cytotoxic effects of the PDT or PDT in combination with EGF-SubA, crystal violet cell viability assay was employed, as described previously.^31^ Two or three independent repeats were included for each experimental group. Briefly, cells were rinsed with PBS, stained for 10 min at room temperature with 0.5% crystal violet in 20% ethanol, and extensively washed with tap water. The violet-stained cells were lysed with 2% SDS solution and transferred to 96-well plate. Absorbance was measured at 595 nm using a microplate reader (ASYS UVM 340, Biochrom, Berlin, Germany). The relative viability was calculated according to the following formula: % viability=[(Ae−Ab)/(Ac−Ab)] × 100%, where Ae is the experimental absorbance, Ab is the background absorbance, and Ac is the absorbance of untreated controls. All experiments were repeated at least three times.

### Flow cytometry

DU-145 cells were trypsinized, pelleted, and washed twice with PBS. The cell suspension was blocked with 1% BSA followed by staining with anti-EGFR chimeric monoclonal antibody (5 μg/ml, Erbitux, Merck, Rahway, NJ, USA) and FITC-conjugated anti-human IgG polyclonal secondary antibody (0.2 μg/ml, Dako, Glostrup, Denmark). Before analysis, cells were washed and resuspended in PBS. The mean fluorescence intensity was analyzed on FACS Scan (Becton Dickinson, San Jose, CA, USA) on a per-cell basis using CellQuest Pro Software Version 5.2.

### Plasmid construction

Human GRP78-coding region was amplified by RT-PCR reaction with primers containing restriction sites for *Xho*I and *Bam*HI on the forward and reverse primer, respectively (Table 1). GRP78 PCR product was digested and cloned into pLVX-IRES-puro vector (Clontech Laboratories, Inc., Saint-Germain-en-Laye, France). Sequence of the pLVX-GRP78-IRES-puro construct was confirmed by DNA sequencing.

### Generation of DU-145 cells stably overexpressing GRP78

DU-145 cell line stably overexpressing GRP78 was generated using second-generation lentiviral system. HEK 293T cells were co-transfected with pLVX-GRP78-IRES-puro plasmid or pLVX-IRES-puro for the generation of a carrier vector control, packaging psPAX2 (Addgene Number 12260) and envelope pMD2.G (Addgene Number 12259) vectors using GeneJuice transfection reagent (Calbiochem, San Diego, CA, USA), according to the manufacturer's protocol. Seventy-two hours after transfection, DU-145 cells were infected with the lentiviruses-containing medium in the presence of 2 μg/ml polybrene (Sigma-Aldrich). Stable cell line was generated using puromycin (Sigma-Aldrich) selection and GRP78 expression was confirmed by means of western blotting.

### Western blotting

Cells were lysed in a lysis buffer (50 mM HEPES pH 7.4, 1,0% Triton X-100, 150 mM NaCl, 10% glycerol, 5 mM EDTA) supplemented with Complete protease inhibitors (Roche, Mannheim, Germany) and phosphatases inhibitors (1 mM sodium ortho-vanadate, 1 mM sodium fluoride, and 1 mM 2-glycerol phosphate). In order to obtain pure cytoplasmic or nuclear fraction, Subcellular Protein Fractionation Kit (Thermo Scientific, Waltham, MA, USA) was employed, according to manufacturer's instructions, with 2 × 10^6^ cells per experimental group. The protein concentration was measured using Bio-Rad Protein Assay (Hercules, CA, USA). Equal amounts of proteins (10–20 *μ*g per well) were separated by SDS-PAGE and transferred to PVDF membrane (Millipore, Billerica, MA, USA) or Protran nitrocellulose membrane (Schleicher and SchuellBioScience, Dassel, Germany). Membranes were blocked and incubated with following primary antibodies (Cell Signaling, Beverly, MA, USA: GRP78–3183, Calnexin–2679, EGFR–4267, P-eIF2*α*–3597, eIF2*α*–2103, CHOP–2895, HDAC2–2540, cytochrome c–2540, cleaved PARP–5625, LC3-I/II–4108, caspase-3–9662, caspase-9–9502; Sigma-Aldrich: *β*-actin–A3854; Calbiochem: *α*-tubulin–CP06; Santa Cruz Biotechnololgy: Hsp90–sc-27987), according to the manufacturer's recommendations. After extensive washing with TBST, membranes were incubated for 40 min with corresponding HRP-linked secondary antibodies (Cell Signaling). The chemiluminescence reaction was developed using self-made reagent (50 mM Tris-HCl pH 8.5, 0.2 mM cumaric acid, 1.25 mM luminol, 0.006% hydrogen peroxide) and visualized with Stella 8300 bio-imager (Raytest, Straubenhardt, Germany). To measure protein loading, membranes were stripped in 0.1 M glycine (pH 2.6) solution and reprobed with anti-*α*-tubulin (Calbiochem) or anti-actin antibody (Sigma-Aldrich).

### RNA interference

Non-targeting human small-interfering RNA (siRNA) and siRNA-targeting human GRP78 or CHOP were purchased from Dharmacon (Thermo Scientific). Transfection was performed via nucleofection technology (Nucleofector II, Lonza, Basel, Switzerland), according to the manufacturer's protocol. Briefly, 2 × 10^6^ of cells were transfected with 20 nM (GRP78) or 50 nM (CHOP) siRNA, seeded on 35-mm plates at a density of 2 × 10^4^/cm^2^ and allowed to attach overnight. Specific downregulation was confirmed by western blotting and quantitative PCR.

### Immunofluorescence

Control and treated DU-145 cells were trypsinized, pelleted, and washed twice with PBS. Next, cells were spun onto the microscopic glass slides using Shandon Cytospin 4 Cytocentrifuge (Thermo Scientific). Cells were fixed in ice-cold methanol for 10 min and washed twice with PBS. Next, cells were blocked with 5% donkey serum for 1 h at 4^o^ C. Afterward, cells were incubated overnight with anti-calnexin primary antibody (Cell Signaling, 1 : 50), washed three times with PBS, and then stained for 30 min with Alexa Fluor 488 Donkey Anti-Rabbit secondary antibody (Molecular Probes, Eugene, OR, USA; 1 : 100). Finally, cell nuclei were stained with DAPI using Vectashield (Vector Laboratories, Burlingame, CA, USA) and were analyzed by fluorescence microscopy (Eclipse TE-2000, Nikon, Tokyo, Japan) with Image ProPlus software (Rockville, MD, USA).

### Electron microscopy

Control and treated cells were trypsynized 8 h after PDT (DU-145 cells) or 16 h after PDT (SW-900 cells), were pelleted and washed twice with PBS. The cell pellets were fixed for 1 h in 2.5% glutaraldehyde and postfixed for 1 h in 1% OsO_4_ both in 0.1 M cacodylate buffer pH 7.4. After dehydration in increasing concentrations of ethanol (50–100%) and in propylene oxide, the material was embedded in Poly/Bed 812 (Polysciences, Inc. Warrington, PA, USA) and cut with a diamond knife on a RMC-type MTXL ultramicrotome on semithin or ultrathin sections. Semithin sections were stained with toluidine blue and inspected in Eclipse E400 (Nikon) light microscope. Ultrathin sections were stained with uranyl acetate and lead citrate, and were examined in a JEM 100S (Jeol, Tokyo, Japan) transmission electron microscope.

### RT-PCR and qPCR

DU-145 or SW-900 cells were rinsed with PBS and lysed in TRIzol Reagent (Invitrogen, Carlsbad, CA, USA). Total cellular RNA was extracted according to the manufacturer's protocol and quantified by NanoDrop 2000c spectrophotometer (Thermo Scientific). Complementary DNA (cDNA) was synthesized from 0.5 *μ*g RNA using oligo(dT) primer and AMV-reverse transcriptase (EURx). PCR for XBP-1 and glyceraldehyde 3-phosphate dehydrogenase was performed using ColorOptiTaq DNA polymerase (EURx, Gdansk, Poland). qPCR was carried out using gene-specific primers (Table 2), cDNAs, and LightCycler 480 SYBRGreen I Master (Roche). The amplification of cDNA was performed using a LightCycler 480 II device (Roche) following the manufacturer's recommendations. In each PCR run, the samples were measured in duplicates and reaction specificity was determined using product melting curves. The results were normalized to two housekeeping genes (*β*2-microglobulin and ribosomal protein L29) and analyzed with LightCycler 480 Software 1.5 (Mannheim, Germany) using Abs Quant/2nd Derivative Max algorithm.

### Apoptosis evaluation

Changes in mitochondrial membrane potential were measured with flow cytometry, using MitoLight dye (Millipore), according to the manufacturer's instructions. Phosphatidylserine plasma membrane externalization was detected with Annexin V Apoptosis Detection Kit (eBioscience, San Diego, CA, USA). Detection of DNA fragmentation was performed with APO-BrdU TUNEL Assay Kit (Life Technologies, Carlsbad, CA, USA) and the total DNA was isolated with GeneMATRIX Cell Culture DNA Purification Kit (Eurx) and subjected to agarose gel electrophoresis and Gel Red (Biotium, Hayward, CA, USA) staining.

### Statistical analysis

Data were presented either in percentages of untreated control or fold-changes over untreated control, with mean±S.D. Mean values were calculated from at least two independent measurements. Student's *t*-test was used for statistical analysis, with significance level set at *P*<0.05 or lower, and is indicated in figure legends. Calcusyn software (Biosoft, Cambridge, England), which is based on Chou–Talalay method for drug combination, was used to evaluate the type of interaction between EGF-SubA cytotoxin and PDT. It calculates the combination index (CI) that allows for the quantitative evaluation of mutual interaction between two therapies (for additive effect CI=1, synergism CI<1, and antagonism CI>1).

## Figures and Tables

**Table 1:** Sequences of primers used for cloning

	**Primer sequence (5′–3′)**
For GRP78*Xho*I	GCGACTCGAGCTGTGGCTGGACTGCCTGC
Rev GRP78*Bam*HI	GCCGGGATCCCTACAACTCATCTTTTTCTGCTGTATCC

Restriction enzymes' cleavage sites are underlined

**Table 2:** Sequences of primers used in PCR and qPCR

**Gene**	**Forward primer sequence (5′**–**3′)**	**Reverse primer sequence (5′**–**3′)**
*XBP-1*	CCTTGTAGTTGAGAACCAGG	GGGGCTTGGTATATATGTGG
*GAPDH*	CCTTCATTGACCTCAACTACATGG	TCACCACCTTCTTGATGTC
*GRP78*	AAGGGGAACGTCTGATTGG	ACGGCAAGAACTTGATGTCC
*EGFR*	CCTGGACAACCCTGACTACC	ACTATCCTCCGTGGTCATGC
*CHOP*	GAGGAGAGAGTGTTCAAGAAGG	TCTGGGAGGTGCTTGTGAC
*B2M*	TAGGAGGGCTGGCAACTTAG	CCAAGATGTTGATGTTGGATAAGA
*RPL29*	CAGCTCAGGCTCCCAAAC	GCACCAGTCCTTCTGTCCTC

## Abbreviations

3-MA: 3-methyladenine
ATF: activation transcription factor
C/EBP: CCAAT/enhancer-binding protein
CHOP: C/EBP homologous protein
EGCG: epigallocatechine gallate
EGF: epidermal growth factor
EGF-SubA: epidermal growth factor fused with subtilase cytotoxin subunit A
EGFR: epidermal growth factor receptor
eIF2*α*: eukaryotic translation initiation factor 2 subunit alpha
ER: endoplasmic reticulum
GRP78: glucose-regulated protein 78
HDAC2: histone deacetylase 2
HSP90: heat-shock protein 90
LC3-I: light chain 3-I, microtubule-associated protein
PDT: photodynamic therapy
PARP: poly (ADP-ribose) polymerase
PI3K: phosphoinositide 3-kinase
PS: photosensitizer
qPCR: quantitative real-time PCR
UPR: unfolded protein response
WA: wortmannin
XBP-1: X-box-binding protein 1
