# Supplementary material for: GRP78-targeting subtilase cytotoxin sensitizes cancer cells to photodynamic therapy
Source: Cell Death Dis. 2013 Jul 25;4(7):e741–. doi: 10.1038/cddis.2013.265 (PMC3730435; doi:10.1038/cddis.2013.265)
Supplement: Supplementary Information [file cddis2013265x1.pdf]

Supplementary Figure legends:

Sup Fig. 1 Induction of GRP78 mRNA by thapsigargin.

After indicated time of incubation with thapsigargin, total RNA was isolated from DU-145, SW-900 and PC-3 cells and reverse transcribed into cDNA. qPCR with LightCycler® Fast Start DNA Master PLUS SYBRGreen I was performed to determine GRP78 mRNA and β_2-microglobulin (B2M) levels. The figure presents mean fold over control change in experimental groups ± SD.

Sup Fig. 2 Evaluation of GRP78 mRNA level by qPCR upon silencing with siRNA.

DU-145 cells treated with GRP78 specific or control siRNA were subjected to *in vitro* PDT as described in Fig. 2. Twenty four hours post PDT the amount of GRP78 mRNA was quantified by qPCR. (-) refers to controls.

Sup Fig. 3 EGFR level at the surface of various cancer cell lines.

EGFR protein level was determined by flow cytometry, as described in Materials and Methods section.

Sup Fig. 4 Photofrin-PDT transiently down-regulates EGFR. DU-145 cells were subjected to *in vitro* PDT at two different doses and analyzed at indicated time points post-PDT. (-) refers to controls.

A. qPCR with specific primers (Table 2) was performed with LightCycler® Fast Start DNA Master PLUS SYBRGreen I. EGFR mRNA amount in each

experimental group was calculated relative to B2M and RPL29. The figure shows fold over untreated control mean values ± SD.

B. EGFR and tubulin (loading control) protein levels in DU-145 total cell lysates estimated by Western blotting. The figure presents a representative result of at least two independent experiments.

C. Detection of EGFR protein level on the surface of DU-145 cells at different time points post-PDT was evaluated by flow cytometry after surface staining of cells with primary antibody (Erbitux) and FITC-labeled secondary anti-human antibody. Left panel shows representative histograms illustrating the level of green fluorescence at indicated times post-PDT, for two PDT doses 4.7 and 7.0 J/cm^2. Right panel shows the quantitative representation of mean fluorescence intensity (mean of two independent measurements ± SD, *$P < 0.05$) relative to untreated control.

Sup Fig. 5 Cytochrome c is released to the cytoplasm of DU-145 cells treated with PDT and PDT+EGF-SubA. DU-145 cells were subjected to *in vitro* PDT and EGF-SubA cytotoxin according to protocol described in Methods section, and 1, 4, 12 and 20 hours post-PDT (7.0 J/cm^2) cells were collected and cytoplasmic fraction was isolated with Subcellular Protein Fractionation Kit (Thermo Scientific). The levels of cytochrome c and Hsp90 (loading control) proteins were evaluated by Western blotting.

Sup Fig. 6 Silencing of CHOP with siRNA

CHOP specific or control siRNA at the final concentration of 50 nM was introduced to DU-145 cells via nucleofection. The cells were seeded into 35 mm dishes and incubated overnight with or without 10 μg/ml Photofrin and 4 pM EGF-SubA cytotoxin. After 24 hours the cells were subjected to *in vitro* PDT and 6 hours later the amount of CHOP mRNA was quantified by qPCR (**A**) or CHOP protein level in whole cell lysates was evaluated by Western blotting (**B**). (-) refers to controls.

Sup Fig. 7 Intermediate and late apoptosis is not enhanced by EGF-SubA cytotoxin in DU-145 and SW-900 cells treated with PDT.

A. Percentage of live, early apoptotic, late stage apoptotic and dead cells was measured with Annexin V Apoptosis Detection Kit (eBioscience). DU-145 and SW-900 cells were collected 20 hours after PDT or actinomycin D treatment. Cells were collected, stained with FITC conjugated Annexin V and propidium iodide according to the protocol and analyzed with flow cytometry. The graph shows percentage of Annexin V+/PI– and Annexin V+/PI+ cells, which corresponds to early apoptotic and late apoptotic cells, respectively. The graph shows mean % from two independent experiments ± SD.

B. GeneMATRIX Cell Culture DNA Purification Kit (Eurx) was used to evaluate DNA fragmentation in DU-145 and SW-900. Control and treated cells were collected 18 hours post PDT or Actinomycin D (1 μg/ml) treatment, and DNA was isolated according to protocol. DNA fragmentation was visualized by agarose gel electrophoresis. (-) refers to controls.

C. Detection of DNA fragmentation for DU-145 cells was also performed with APO™-BrdU TUNEL Assay Kit (Life Technologies). DU-145 cells were collected 16 hours post-PDT or actinomycin D (1 µg/ml) treatment, and DNA-labeling was performed according to the manufacturer's protocol.

Sup Fig. 8 DU-145 cells treated with EGF-SubA and PDT do not undergo necrosis

A. Lactate dehydrogenase (LDH) release to cell culture medium was measured with colorimetric assay (Sigma). DU-145 cells were subjected to *in vitro* PDT in the presence or absence of 4 pM EGF-SubA. The culture medium was collected 4, 8, and 16 hours post-PDT, and, after removal of media, the cells were solubilized with lysis buffer. LDH activity was determined in supernatants and cell lysates according to manufacturer's protocol. The % of LDH released to the culture medium was calculated according to the formula: %LDH release = LDH activity in supernatant/(LDH activity in supernatant + LDH activity in total cells) × 100%. Mean values from three independent measurements are shown, and SD values, which range from 5 to 25 % of the mean, are omitted for the clarity of the figure. The differences in LDH release for each experimental time point are statistically insignificant. (-) refers to controls.

B. Cell viability in the absence (control) and presence of 50 µM necrostatin-1 was determined by crystal violet staining, as described in Fig.4. The graph shows mean % from two independent experiments ± SD.

Sup Fig. 9 Autophagy markers are not observed in DU-145 cells

A. DU-145 cells were subjected to *in vitro* PDT in the presence or absence of 4 pM EGF-SubA or to 50 µM rapamycin treatment. For indicated samples,

chloroquine (50 μM) was added to the culture medium 2 h prior to treatment and maintained for additional 16h until the whole cell lysates were collected and analyzed by Western blotting for the level of LC3-I and LC3-II. HeLa and A549 were incubated with or without 50 μM chloroquine for 18 h. (-) refers to controls.

B. DU-145 cells were subjected to *in vitro* PDT in the presence of 4 pM EGF-SubA. For indicated samples, 2 hours before PDT and immediately post PDT 3-MA was added to the culture medium at the final concentration of 2 mM or 4 mM. Cell viability was determined by crystal violet staining, 16 h and 24 h post-PDT.

Sup Fig. 10 Representative ultrastructural changes in cells treated with PDT and EGF-SubA cytotoxin

PDT and EGF-SubA treated DU-145 cells were prepared for electron microscopic observation as described in Fig. 7. Scale bars represent 5 μm.

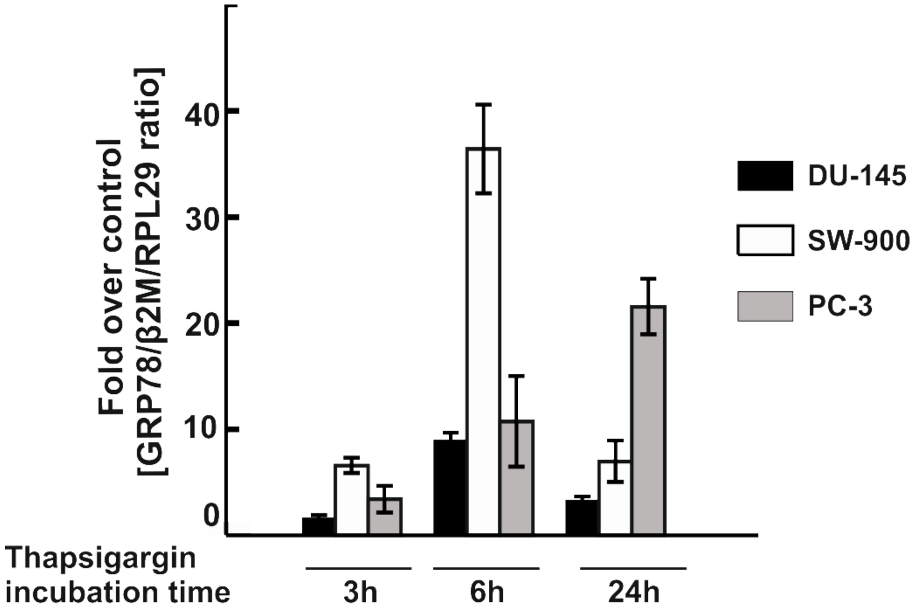
Fold over control
[GRP78/β2M/RPL29 ratio]
40
30
20
10
0
DU-145
SW-900
PC-3
Thapsigargin
incubation time
3h
6h
24h

Sup Fig.1

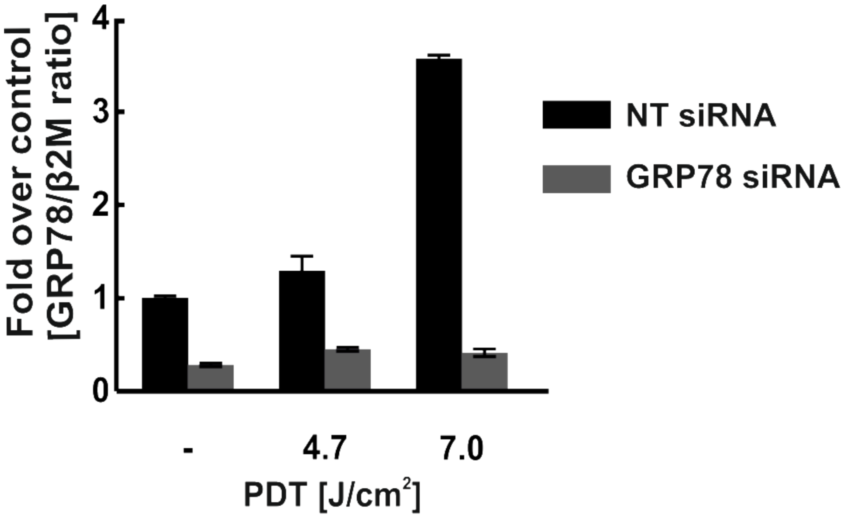

Fold over control
[GRP78/β2M ratio]
4
3
2
1
0
NT siRNA
GRP78 siRNA
-
4.7
7.0
PDT [J/cm2]

Sup Fig.2

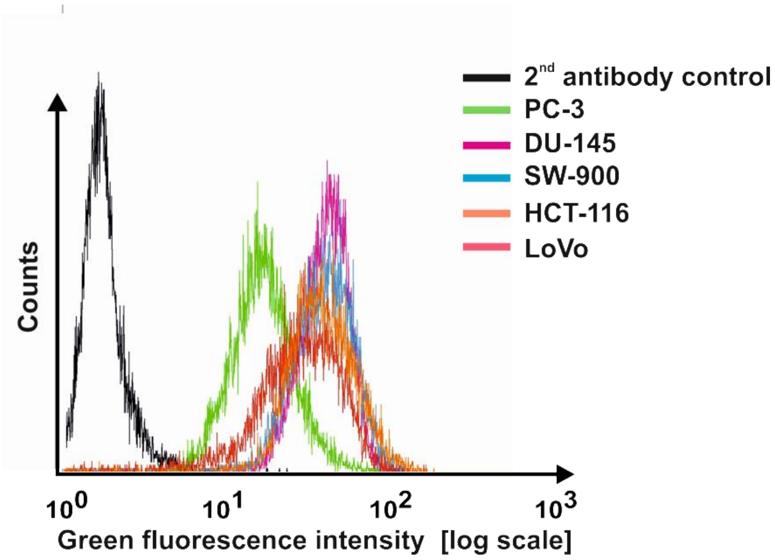
2nd antibody control
PC-3
DU-145
SW-900
HCT-116
LoVo
Counts
10^0
10^1
10^2
10^3
Green fluorescence intensity [log scale]

Sup Fig.3

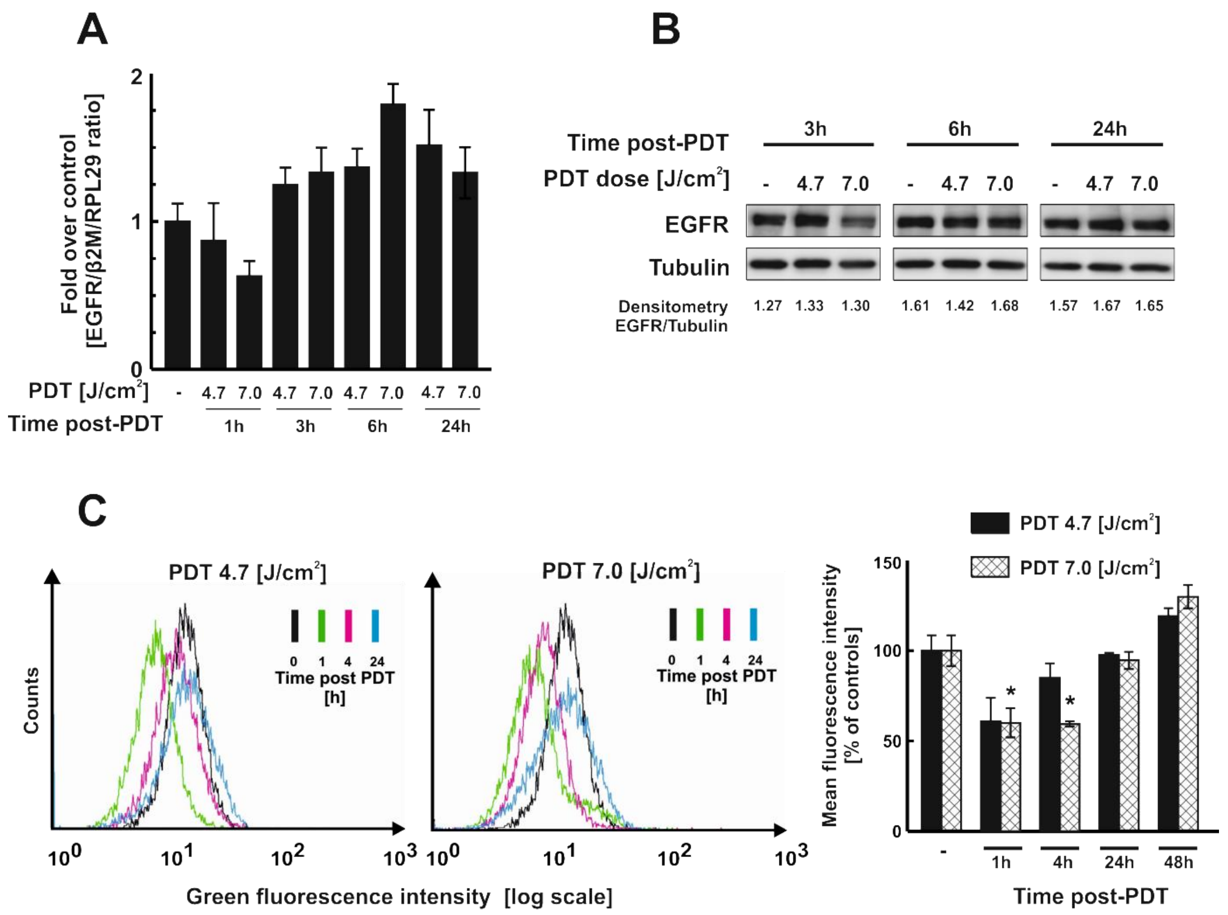
A
Fold over control [EGFR/β2M/RPL29 ratio]
2
1
0
PDT [J/cm2]
-
4.7
7.0
Time post-PDT
1h
3h
6h
24h
B
Time post-PDT
3h
6h
24h
PDT dose [J/cm2]
EGFR
Tubulin
Densitometry EGFR/Tubulin
1.27 1.33 1.30 1.61 1.42 1.68 1.57 1.67 1.65
C
PDT 4.7 [J/cm2]
PDT 7.0 [J/cm2]
Counts
0 1 4 24
Time post PDT [h]
Green fluorescence intensity [log scale]
Mean fluorescence intensity [% of controls]
150
100
50
0
1h
4h
24h
48h
Time post-PDT

Sup Fig. 4

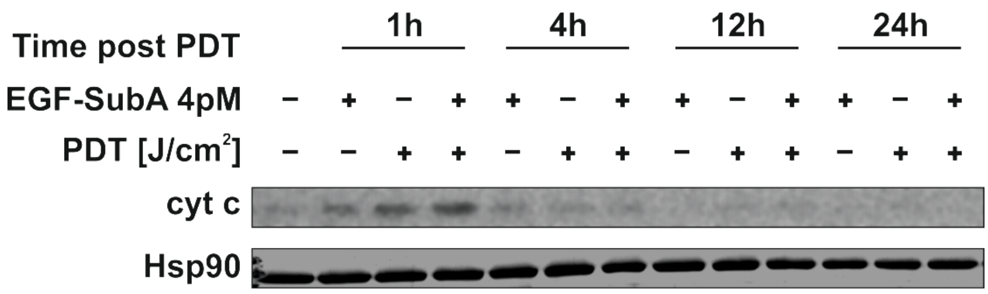

Sup Fig. 5

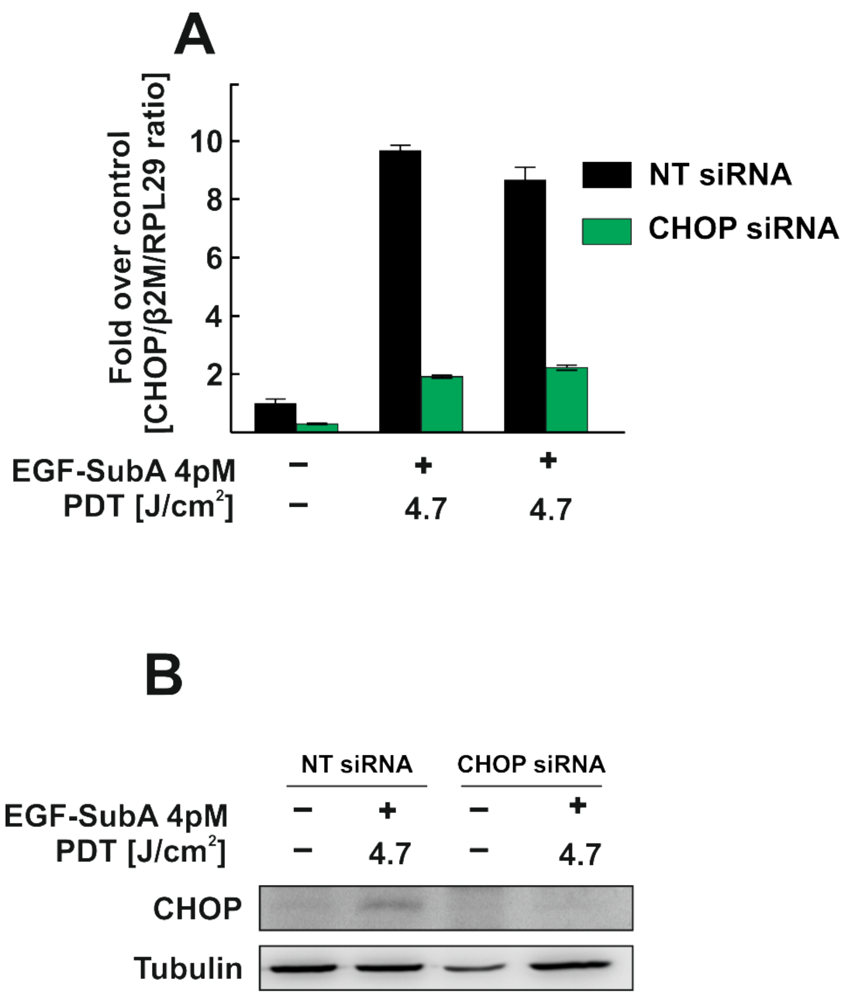
A
Fold over control
[CHOP/β2M/RPL29 ratio]
10
8
6
4
2
NT siRNA
CHOP siRNA
EGF-SubA 4pM
PDT [J/cm2]
4.7
4.7
B
NT siRNA
CHOP siRNA
EGF-SubA 4pM
PDT [J/cm2]
4.7
4.7
CHOP
Tubulin

Sup Fig. 6

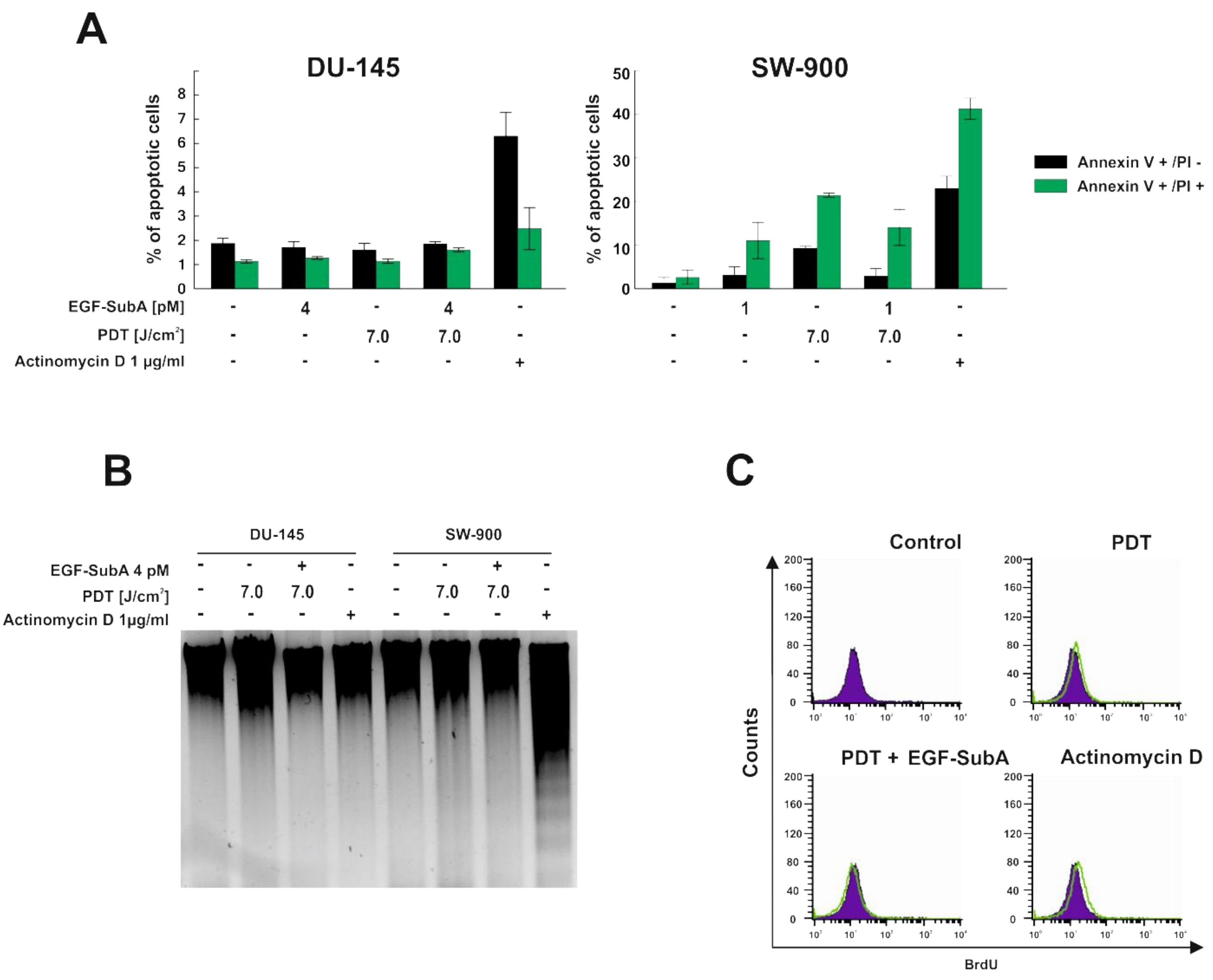
A
DU-145
SW-900
% of apoptotic cells
Annexin V + /PI -
Annexin V + /PI +
EGF-SubA [pM]
PDT [J/cm²]
Actinomycin D 1 µg/ml
B
DU-145
SW-900
EGF-SubA 4 pM
PDT [J/cm²]
Actinomycin D 1µg/ml
C
Control
PDT
PDT + EGF-SubA
Actinomycin D
Counts
BrdU

Sup Fig. 7

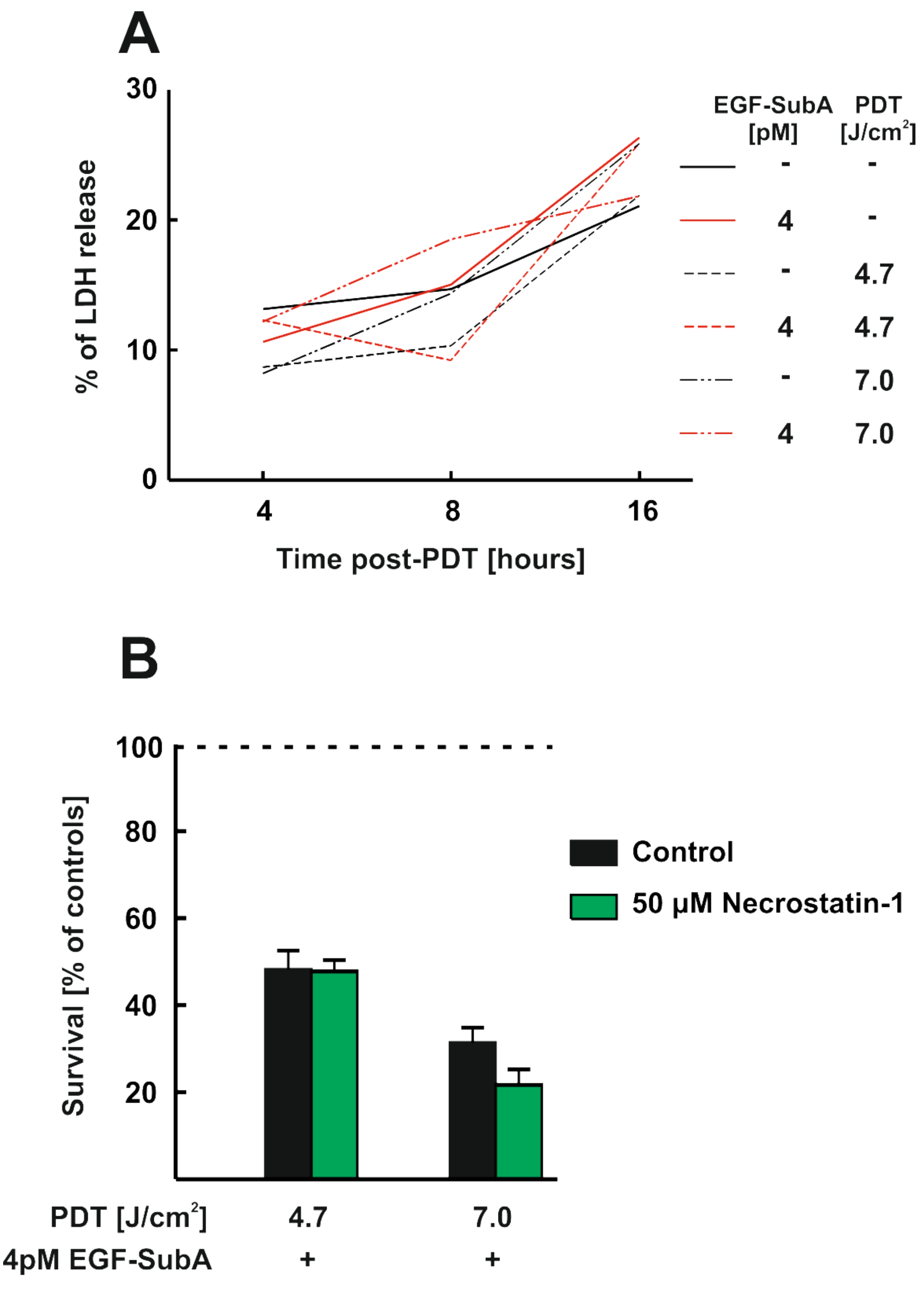
A
30
20
10
0
% of LDH release
4
8
16
Time post-PDT [hours]
EGF-SubA [pM]
PDT [J/cm2]
- -
4 -
- 4.7
4 4.7
- 7.0
4 7.0
B
100
80
60
40
20
Survival [% of controls]
Control
50 μM Necrostatin-1
PDT [J/cm2]
4.7
7.0
4pM EGF-SubA
+
+

Sup Fig. 8

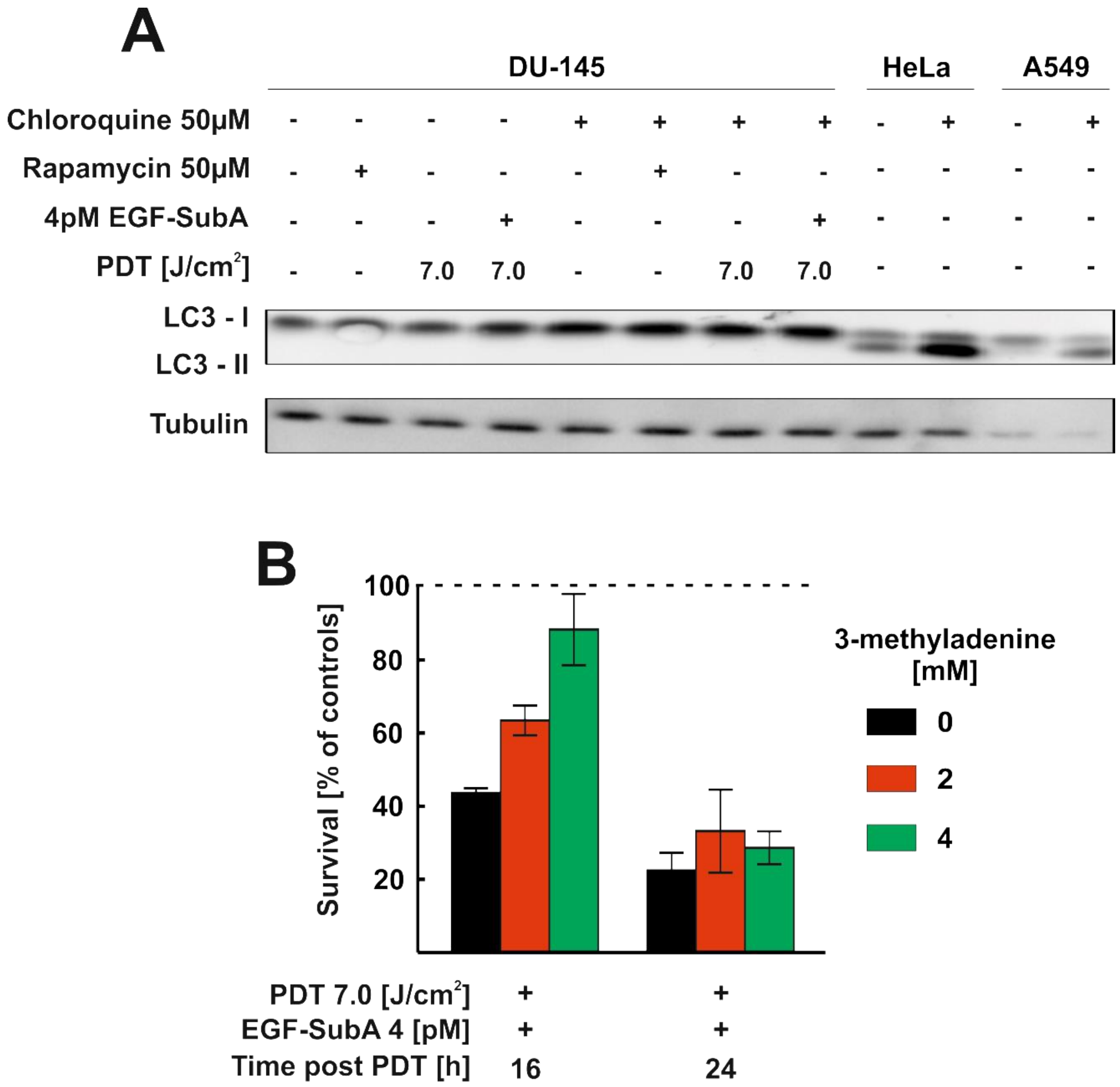
A
DU-145
HeLa
A549
Chloroquine 50µM
Rapamycin 50µM
4pM EGF-SubA
PDT [J/cm²]
7.0
LC3 - I
LC3 - II
Tubulin
B
Survival [% of controls]
100
80
60
40
20
3-methyladenine [mM]
0
2
4
PDT 7.0 [J/cm²]
EGF-SubA 4 [pM]
Time post PDT [h]
16
24

Sup Fig. 9

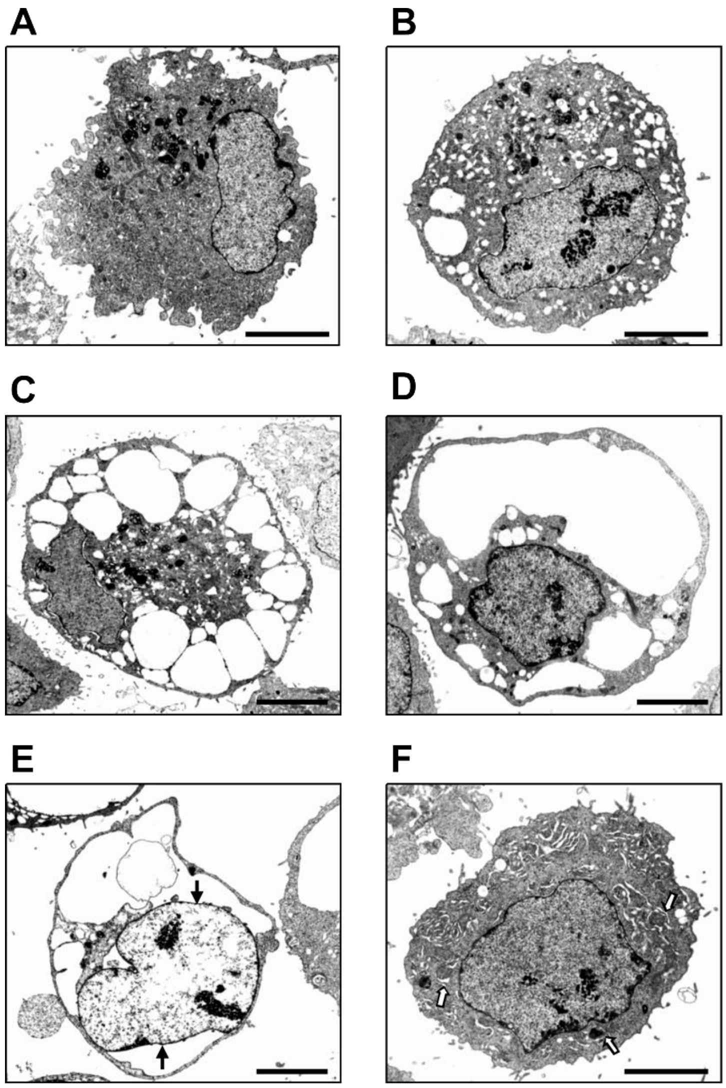

Sup Fig. 10
